# Power-Independent Microwave Photonic Instantaneous Frequency Measurement System

**DOI:** 10.3390/s25144382

**Published:** 2025-07-13

**Authors:** Ruiqiong Wang, Yongjun Li

**Affiliations:** Information and Navigation College, Air Force Engineering University, Xi’an 710003, China; tz_228@163.com

**Keywords:** instantaneous frequency measurement (IFM), power-independent, microwave photonics, optical tunable delay line (OTDL)

## Abstract

The ability to perform instantaneous frequency measurement (IFM) of unknown microwave signals holds significant importance across various application domains. This paper presents a power-independent microwave photonic IFM system. The proposed system implements frequency measurement through the construction of an amplitude comparison function (ACF) curve, achieved by introducing a frequency-dependent time delay via an optical tunable delay line (OTDL) for the signal under test (SUT). System simulation demonstrates the measurement capability across a wide bandwidth of 0.1–40 GHz with high precision, exhibiting frequency errors ranging from −0.03 to 0.04 GHz. The scheme also maintains consistent performance under varying input power levels. Key implementation aspects, including single-sideband modulation selection and system extension methods, are analyzed in detail to optimize measurement accuracy. Notably, the proposed architecture features a simple and compact design with excellent integration potential. These characteristics, combined with its wide operational bandwidth and high measurement precision, make this approach particularly suitable for demanding applications in electronic reconnaissance and communication.

## 1. Introduction

In modern electronic reconnaissance and communication, the first step in signal detection and measurement is usually instantaneous frequency measurement (IFM) [1]. However, traditional microwave measurement methods have a limited measurement range, typically requiring down-conversion processing for high-frequency signals, and are susceptible to electromagnetic interference. Microwave photonics aims to use optical technology to generate, transmit, control, and process microwave signals. It has advantages such as a large bandwidth, a small size, and anti-electromagnetic interference [2,3,4]. In recent years, IFM technology based on microwave photonics has attracted great attention [5,6].

There are three kinds of photonic-assisted IFM techniques, which are based on frequency–time mapping (FTTM) [7,8,9,10,11,12,13,14], frequency–space mapping, and frequency–power mapping. FTTM can be achieved by establishing the relationship between the frequency of an unknown microwave signal and the electrical delay, and the electrical delay can be obtained using dispersion delay [7] or frequency-shift cyclic delay lines [8,9]. These schemes have the ability to measure multi-tone frequencies, but the measurement resolution is limited. Recently, optical sideband scanning, frequency down conversion and intermediate frequency envelope detection have been used to achieve FTTM [10,11,12]. These methods can be flexibly adjusted, but the frequency measurement range is limited. Frequency–space mapping is mostly based on channelization methods [15,16,17], which can achieve frequency measurement by detecting the power distribution of different channels. However, this method is usually complicated in structure, and it is difficult to achieve a large bandwidth and high precision at the same time.

In the measurement of single-frequency signals, the frequency–power mapping scheme has the advantages of a wide measurement range, high accuracy, and great application potential. Frequency–power mapping can be further divided into two types, frequency–radio frequency (RF) power mapping and frequency–optical power mapping, both of which focus on the construction of amplitude comparison functions (ACFs).

In frequency–RF power mapping, the microwave power ratio of two parallel channels can be achieved through dispersion elements [18,19,20,21], optical mixing units [22], optical filters [23], or optical processors [24], thereby constructing an ACF. The above systems typically require multiple lasers, modulators, or dispersive media, which increase the complexity and cost of the system. For frequency–RF power mapping, a tunable optical delay line and a 90-degree hybrid are used in [25] to establish two ACFs that convert frequency information into optical power ratios. In refs. [26,27], the signal to be measured is modulated on two optical carriers whose wavelengths are set at special points in the spectral response of the sinusoidal filter. The frequency of the microwave signal is obtained by comparing the optical power output of the two wavelength channels. In ref. [28], the linear frequency response of wavelength division multiplexing is used to construct frequency–power mapping for IFM, but the wavelength drift of the laser source and the spectral response shift of the filter will cause measurement errors. In reference [29], an electrical delay and polarization control are used to achieve frequency–optical power mapping, but the system is polarization-sensitive. In ref. [30], two phase modulators and a Sagnac loop are used to construct two frequency-dependent DC power sources, thereby constructing an ACF. Reference [31] relies on employing two signal time delays within a microwave photonic structure to create two low-frequency signal phase differences.

An IFM scheme based on a Mach–Zehnder interferometer (MZI) structure is proposed in this paper. The scheme has the following advantages. First, the ACF curve is constructed by the ratio of two signal amplitudes, which can improve the measurement accuracy; second, the scheme can achieve high-precision measurement with a large and unambiguous bandwidth by introducing multiple delays through link expansion; third, the scheme is not affected by the power of the signal under test (SUT), which makes it more practical in applications; fourth, the scheme is simple in structure and does not contain polarization devices, so it has great prospects in electronic reconnaissance, electronic countermeasures, and other applications. Our simulation results show that this scheme can achieve high precision measurement in a wide bandwidth of 0.1–40 GHz, and the measurement error is only −0.03 to 0.04 GHz.

The structure of this paper is organized as follows: Section 2 will explain the principles of the proposed scheme. Section 3 will present the simulation results obtained from the scheme. Section 4 will discuss the performance of the scheme. Finally, Section 5 will conclude the paper and explore the potential for future applications.

## 2. Principle

**Figure 1:**
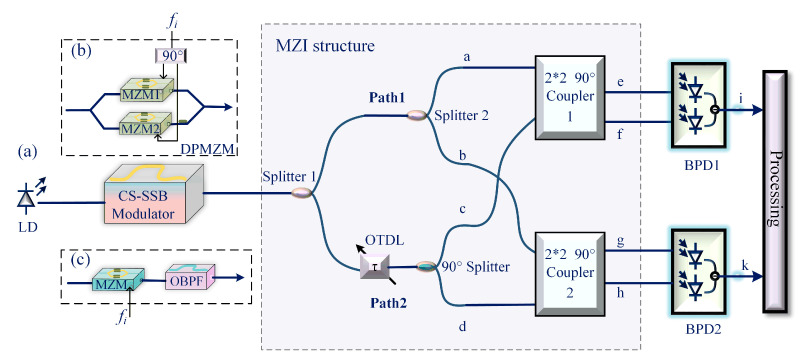
Principle of (**a**) the proposed system, (**b**) CS-SSB based on DPMZM, (**c**) CS-SSB based on filter.

The microwave photonic IFM measurement system proposed in this paper is shown in Figure 1a. A continuous light wave is generated by a laser diode (LD), and then carrier-suppressed single-sideband (CS-SSB) modulation is applied to the optical carrier. There are two implementation methods for CS-SSB modulation, as shown in Figure 1b,c; in Figure 1b, CS-SSB modulation is achieved based on a dual-parallel Mach–Zender modulator (DPMZM) and a 90° power divider, and in Figure 1c, MZM is biased at the minimum point to achieve carrier-suppressed double-sideband modulation, while optical a bandpass filter (OBPF) is used to filter out one of the sidebands. Then, the CS-SSB signal is transmitted to the MZI structure. In the MZI structure, the CS-SSB signal is first evenly divided into two paths by power splitter 1. In path 1, the optical signal is equally divided into two parts by power splitter 2, as shown by two points, a and b, in Figure 1a. The optical signal in path 2 is first transmitted to the optical tunable delay line (OTDL) to introduce a frequency-dependent phase shift and then injected into the 90° power divider to be equally divided into two parts with a phase difference of 90°, as shown in points c and d in Figure 1a. Then, the optical signals represented by points a and c are transmitted to the two input ports of 2 × 2 90° coupler 1, and the two output signals are shown at points e and f in Figure 1a. Finally, the two output signals are sent to a balanced photodetector (BPD1) for photoelectric detection. Similarly, in path 2, the optical signals represented by points b and d are transmitted to the two input ports of 2 × 2 90° coupler 2; then, the two output signals (points g and h) are sent to BPD2 for photoelectric detection.

The optical carrier can be written as *E*_in_(*t*) = *E*_c_ exp(*j*2*πf_c_t*), where *f_c_* and *E*_c_ are the frequency and amplitude. The RF signal to be measured is expressed as *V_i_* cos(2*πf_i_t*), where *f_i_* and *V_i_* are the frequency and amplitude of the SUT. When the RF signal is electro-optic-modulated using the structure shown in Figure 1b, the two sub-modulators are biased at the minimum point, and the main modulator is biased at the quadrature point; then, the output of the CS-SSB modulation module is represented as

(1) $$E_{CS-SSB}(t)\propto \frac{\alpha_{DPMZM}}{\sqrt{2}}E_{c}jJ_{1}\left(m\right)\exp\left[j2\pi \left(f_{c}+f_{i}\right)t\right]$$

where $\alpha_{DPMZM}$ represents the optical insertion loss of the DPMZM, *m = πV_i_/V_π_* is the modulation index of the DPMZM, *V_π_* is the half-wave voltage, and *Jn* (·) represents the nth-order Bessel function of the first kind. Considering the limited modulation index, the higher-order Bessel functions are ignored. If the structure shown in Figure 1c is used, and the MZM works at the minimum point, then the output of the CS-SSB modulation module is represented as

(2) $$E_{CS-SSB}(t)\propto E_{c}\alpha_{MZM}\alpha_{OBPF}jJ_{1}\left(m\right)\exp\left[j2\pi \left(f_{c}+f_{i}\right)t\right]$$

where $\alpha_{MZM}$ and $\alpha_{OBPF}$ represent the optical insertion loss of the MZM and the OBPF, respectively.

**Figure 2:**
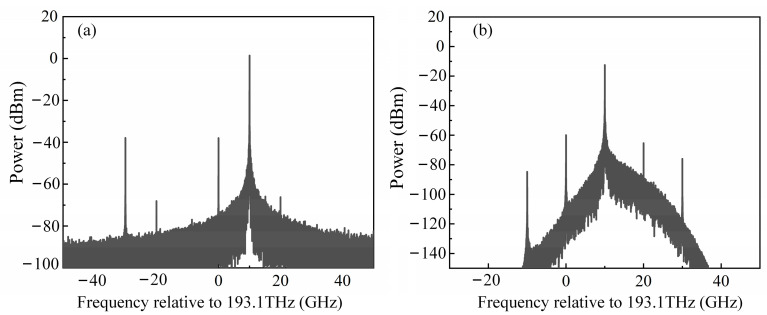
CS-SSB modulation based on (**a**) DPMZM and (**b**) OBPF.

To select a more suitable single-sideband implementation scheme for this system, simulations of the two cases shown in Figure 1b,c were conducted using the VPI Transmission Maker 11.1 simulation software. The simulation results for both methods are shown in Figure 2. In the CS-SSB structure based on DPMZM (Figure 2a), the optical Hilbert transform is constructed by using a 90° electric phase shifter, and the CS-SSB output is finally realized. Because the extinction ratio of the modulator is limited, the carrier cannot be completely suppressed, and the carrier–side ratio is about −40 dB. In addition to the expected +1 order sideband, there is also a −3 order sideband, but the power is low and can generally be ignored. Figure 2b shows the OBPF-based CS-SSB structure, where the filter parameters are set according to the EXFO-XTM-50 device. The MZM is biased at the minimum point, and the carrier-to-sideband ratio exceeds −40 dB. When the bandwidth of the filter is narrow enough, it can even completely filter out the optical carrier. In addition, the spectrum is relatively pure, and the noise level is also low. However, the operating bandwidth of the system will be limited, especially in the low frequency range. When the frequency is too low, it is likely to filter out other sidebands at the same time. Therefore, in order to achieve a large bandwidth measurement, a CS-SSB based on a DPMZM is selected in this paper.

Then, the CS-SSB optical signal is equally divided into two channels through optical power splitter 1, in which the path 1 signal is transmitted to optical power splitter 2. At this time, the output optical signal is shown by two points, a and b, in Figure 1a. After passing through the OTDL, the signal in path 2 is divided into two equal paths with a phase difference of 90°, as shown at points c and d. At this point, the four optical signals can be represented as

(3) $$\left[\begin{array}{l} E_{a}\left(t\right)\\ E_{b}\left(t\right)\\ E_{c}\left(t\right)\\ E_{d}\left(t\right) \end{array}\right]=\left[\begin{array}{l} \frac{E_{CS-SSB}\left(t\right)}{2}\\ \frac{E_{CS-SSB}\left(t\right)}{2}\\ \frac{E_{CS-SSB}\left(t\right)}{2}\exp\left[-j\left(\varphi +\frac{\pi }{2}\right)\right]\\ \frac{E_{CS-SSB}\left(t\right)}{2}\exp\left(-j\varphi \right) \end{array}\right]$$

where *φ* = 2*πτf_i_* is the frequency-dependent phase shift introduced by OTDL, and *ꞇ* is the time delay introduced by the OTDL. Subsequently, the optical signals of path a and path c are sent to the two input ports of 2 × 2 90° coupler 1. The transmission matrix of the 2 × 2 90° coupler is as follows:

(4) $$H=\frac{1}{2}\left[\begin{array}{l} 1\\ j \end{array}\right.\left.\begin{array}{l} j\\ 1 \end{array}\right]$$

The two outputs of 2 × 2 90° coupler 1 can be expressed as

(5) $$\left[\begin{array}{l} E_{e}\left(t\right)\\ E_{f}\left(t\right) \end{array}\right]=\frac{1}{2}\left[\begin{array}{l} E_{a}\left(t\right)+E_{c}\left(t\right)\cdot \exp\left(j\frac{\pi }{2}\right)\\ E_{a}\left(t\right)\cdot \exp\left(j\frac{\pi }{2}\right)+E_{c}\left(t\right) \end{array}\right]=\frac{1}{2}\left[\begin{array}{l} \frac{E_{CS-SSB}\left(t\right)}{2}+\frac{E_{CS-SSB}\left(t\right)}{2}\exp\left[-j\left(\varphi +\frac{\pi }{2}\right)\right]\cdot \exp\left(j\frac{\pi }{2}\right)\\ \frac{E_{CS-SSB}\left(t\right)}{2}\cdot \exp\left(j\frac{\pi }{2}\right)+\frac{E_{CS-SSB}\left(t\right)}{2}\exp\left[-j\left(\varphi +\frac{\pi }{2}\right)\right] \end{array}\right]$$

Then, the two optical signals enter BPD1 for photodetection; the output photocurrent can be expressed as

(6) $$i_{BPD1}(t)=\eta \left\{{\left|E_{e}(t)\right|}^{2}-{\left|E_{f}(t)\right|}^{2}\right\}=\frac{1}{2}\eta E_{c}^{2}\alpha_{DPMZM}^{2}J_{1}^{2}\left(m\right)\cos\varphi$$

where *η* is the responsiveness of the BPD. Similarly, the b-path optical signal and d-path optical signal are fed into the two input ports of 2 × 2 90° coupler 2, and the outputs can be expressed as

(7) $$\left[\begin{array}{l} E_{g}\left(t\right)\\ E_{h}\left(t\right) \end{array}\right]=\left[\begin{array}{l} E_{b}\left(t\right)+E_{d}\left(t\right)\cdot \exp\left(j\frac{\pi }{2}\right)\\ E_{b}\left(t\right)\cdot \exp\left(j\frac{\pi }{2}\right)+E_{d}\left(t\right) \end{array}\right]=\left[\begin{array}{l} \frac{E_{CS-SSB}\left(t\right)}{2}+\frac{E_{CS-SSB}\left(t\right)}{2}\exp\left(-j\varphi \right)\cdot \exp\left(j\frac{\pi }{2}\right)\\ \frac{E_{CS-SSB}\left(t\right)}{2}\cdot \exp\left(j\frac{\pi }{2}\right)+\frac{E_{CS-SSB}\left(t\right)}{2}\exp\left(-j\varphi \right) \end{array}\right]$$

Then, the two optical signals output by 2 ×2 90° coupler 2 are transmitted to BPD2 for photoelectric detection:

(8) $$i_{BPD2}(t)=\eta \left\{{\left|E_{g}(t)\right|}^{2}-{\left|E_{h}(t)\right|}^{2}\right\}=\frac{1}{2}\eta E_{c}^{2}\alpha_{DPMZM}^{2}J_{1}^{2}\left(m\right)\sin\varphi$$

According to Equations (6) and (8), an ACF can be constructed to measure the frequency of the SUT, expressed as

(9) $$ACF=\frac{i_{BPD2}(t)}{i_{BPD1}(t)}=\tan\varphi =\tan(2\pi \tau f_{i})$$

From (9), It can be seen that the frequency of the SUT can be obtained by measuring the amplitude of the output DC signal of the BPD. It can also be seen from Equation (9) that since the ratio is used to construct the ACF, the amplitude can avoid the influence of other factors, such as RF signal power, BPD responsivity, etc.

## 3. Simulation Results

**Figure 3:**
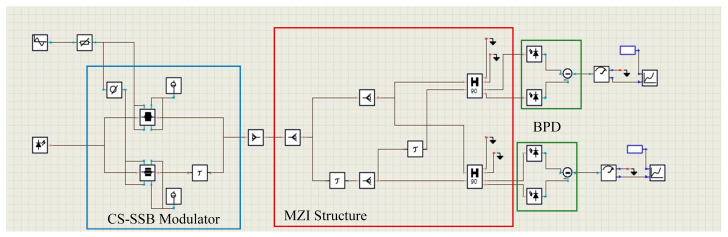
Schematic diagram of simulation link construction.

The VPI Transmission Maker simulation platform was built as shown in Figure 3. In order to be close to the actual experiment, the parameters are set according to the actual experiment in the simulation. For example, the DPMZM is set according to the FTM7961 device (Fujitsu (Tokyo, Japan)). The half-wave voltage of the modulator is influenced by factors such as the material, waveguide structure, operating frequency, and temperature. A high half-wave voltage can negatively impact signal quality and modulation efficiency. In the experiments, the efficiency of the modulator is frequency-dependent, and thus, the half-wave voltage may vary at different frequency points. Taking the four frequency points of 10 GHz, 20 GHz, 30 GHz, and 40 GHz as an example, the half-wave voltages are about 3.85 V, 4.3 V, 4.67 V, and 4.94 V respectively. Therefore, in the simulation, different half-wave voltages need to be set according to different frequency bands; for example, the half-wave voltage is set to 4.3 V at 0.1–18 GHz and 4.94 V at 26–40 GHz. Meanwhile, a phase error of 5 degrees is introduced into the 90° coupler, and an amplitude error of 0.5 dB and a phase error of 3 degrees are introduced into the BPD module. The actual devices and parameter designs referenced in the simulation are shown in Table 1.

A continuous optical carrier with a power of 16 dBm and a frequency of 193.1 THz is generated from the LD. Then, the optical carrier is injected into the CS-SSB modulator, which contains a DPMZM and a 90° power divider. The SUT is modulated by the optical carrier in the CS-SSB modulator; the power of the SUT is 5 dBm. Then, the SSB signal is transmitted to splitter 1 and divided into two power paths, as shown in Figure 1. In path 1, the optical signal is transmitted to splitter 2 and divided into two equal paths, as shown in path a and path b. The optical signal in path 2 first introduces a delay related to the frequency of the SUT through the OTDL, and the delayed signal is divided into two channels by a 90° splitter; as shown in paths c and d, a 90° phase difference is introduced into paths c and d. Then, the two optical signals from path a and path c are injected into the two input ports of 2 × 2 90° coupler 1. Finally, the two output signals from 2 × 2 90° coupler 1 are injected into BPD1 for photoelectric detection. Similarly, the two optical signals of path b and path d are injected into the two input ports of 2× 2 90° coupler 2, and the two output signals of 2 × 2 90° coupler 2 are injected into the BPD2; the responsivity of the BPD is 0.6 A/W.

**Figure 4:**
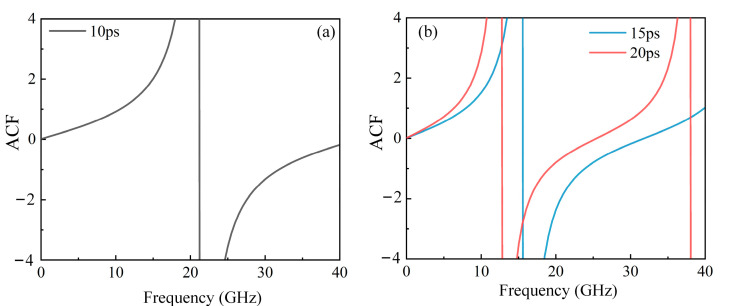
ACF curves under different delays (**a**) delay of 10 ps (**b**) delay of 15 ps and 20 ps.

First, the ACF curves of the scheme are validated. The delay of the OTDL is set to 10 ps, the frequency of the SUT is changed from 0.1 GHz to 40 GHz, the output amplitudes of BPD1 and BPD2 are saved, and the corresponding ACF curve is calculated, as shown in Figure 4a. From Figure 4a, it can be seen that the scheme can achieve a frequency measurement range exceeding 30 GHz with a delay of 10 ps. Similarly, the delay is changed to 15 ps and 20 ps, and the ACFs obtained by repeating the above operations are shown in Figure 4b; it can be seen that different delays can achieve different frequency measurement ranges.

**Figure 5:**
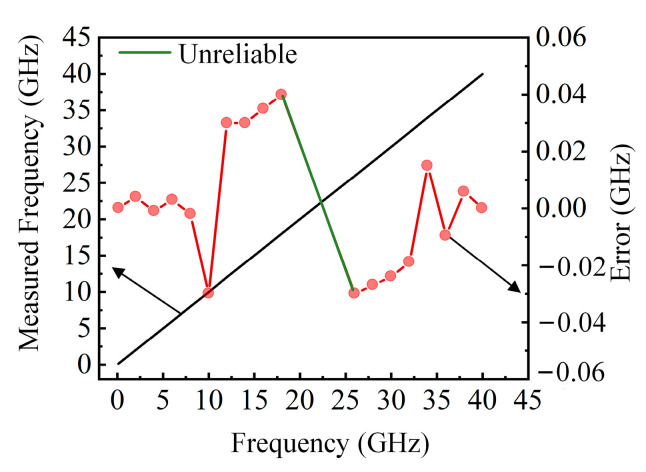
Frequency measurement results and measurement errors.

In order to verify the frequency measurement performance of the proposed scheme, the delay of the OTDL is set to 10 ps, and the SUT signal is changed from 0.1 GHz to 40 GHz with a step of 2 GHz. The ratio of the output amplitudes at each frequency is calculated and compared with the ACF curve to obtain the measurement value and error; the results are shown in Figure 5. As seen in Figure 5, when the delay is 10 ps, the frequency measurement range is approximately 0.1–18 GHz and 26–40 GHz, and the measurement error is between −0.03 and 0.04 GHz, as shown by the red dots in Fig. 5. A maximum relative measurement error of 0.3% is obtained across the entire frequency range. Due to significant measurement errors in the 18–26 GHz range, this interval is defined as the “unreliable interval”. The measurement range exceeds 30 GHz, and the results have proven that the scheme can achieve high-precision measurement in a large frequency range.

**Figure 6:**
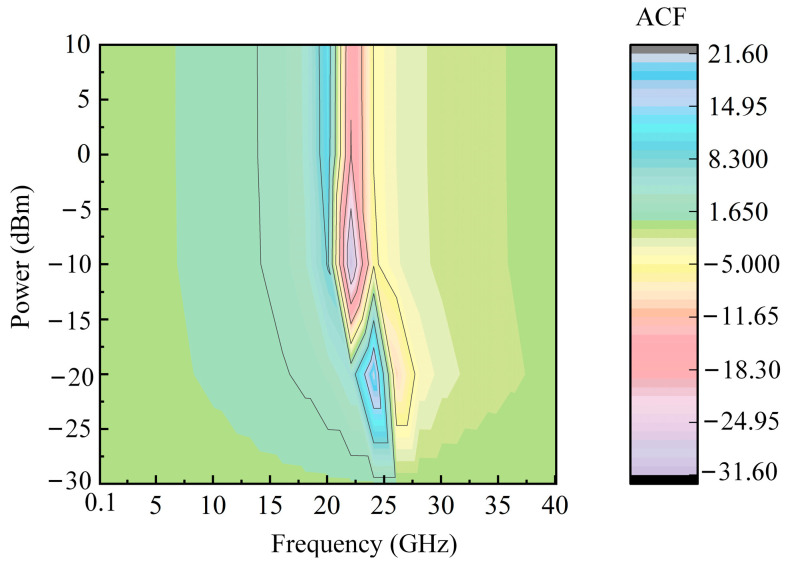
The relation of ACF curve with the frequency and power of SUT signal.

To verify the influence of SUT power on frequency measurement results, the relationship of the ACF curve with the frequency and power of the SUT signal is simulated and plotted in Figure 6. In the simulation, the delay is set to 10 ps, and the SUT frequency is changed from 0.1 GHz to 40 GHz. At the same time, the SUT power is changed from −30 dBm to 10 dBm. The corresponding ACF value is calculated, and a three-dimensional curve is drawn. From Figure 6, it can be seen that the ACF curve can maintain monotonicity over a large frequency range under different SUT powers; especially when the SUT power is greater than −20 dBm, the ACF curve almost does not change with power changes. The power tunability of the scheme is proved.

**Figure 7:**
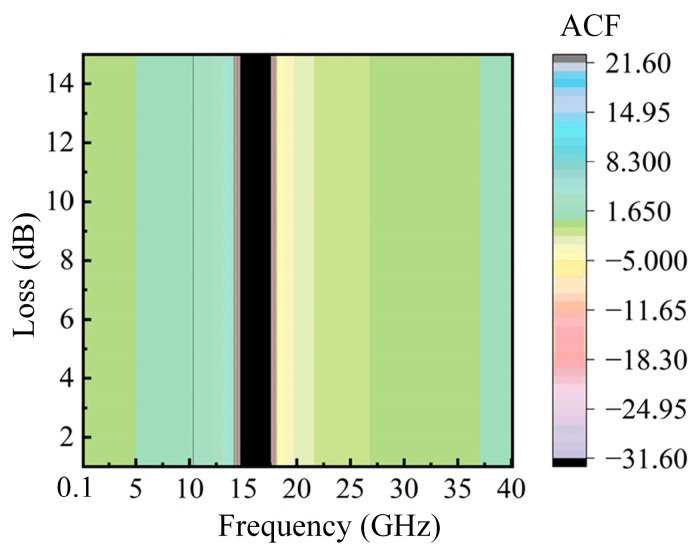
The relation of ACF curve with the frequency and loss of the modulator.

The impact of the insertion loss of the modulator on the system is also verified. In the simulation, a delay of 15 ps is set, and the modulator loss is varied from 1 dB to 15 dB, while the SUT frequency is changed from 0.1 GHz to 40 GHz. The resulting ACF three-dimensional curve is shown in Figure 7. It can be observed from the figure that as the insertion loss increases, the trend of the ACF curve remains unchanged, which confirms that the performance of the system is independent of insertion loss.

## 4. Discussion

### 4.1. Discussion on Path Lengths

In the above simulation, the four paths of a, b, c, and d in Figure 1 are set to have the same length, and the different path lengths are briefly discussed below. First, the length of path a is the same as that of path b, and the length of path c is the same as that of path d. Assuming path a is L1 (m) longer than path c, then paths a and b introduce a phase shift, $\varphi_{L1}$, related to L1, which is also related to the frequency to be measured. At this point, (3) can be rewritten as

(10) $$\left[\begin{array}{l} E_{a}\left(t\right)\\ E_{b}\left(t\right)\\ E_{c}\left(t\right)\\ E_{d}\left(t\right) \end{array}\right]=\left[\begin{array}{l} \frac{E_{CS-SSB}\left(t\right)}{2}\exp\left(-j\varphi_{L1}\right)\\ \frac{E_{CS-SSB}\left(t\right)}{2}\exp\left(-j\varphi_{L1}\right)\\ \frac{E_{CS-SSB}\left(t\right)}{2}\exp\left[-j\left(\varphi +\frac{\pi }{2}\right)\right]\\ \frac{E_{CS-SSB}\left(t\right)}{2}\exp\left(-j\varphi \right) \end{array}\right]$$

The ACF expression derived from (10) is

(11) $$\begin{array}{l} ACF=\tan(\varphi_{L1}-\varphi )=\tan\left[2\pi f_{i}(\tau_{L1}-\tau )\right]\\ \tau_{L1}=nL_{1}/c \end{array}$$

where *n* is the core refractive index, and *c* is the speed of light in a vacuum. It can be seen that a monotonic ACF curve can still be obtained at this time

Then, assuming that paths b, c, and d are equal in length, and path a is L2 (m) longer than the other paths, the resulting ACF curve can be expressed as

(12) $$\begin{array}{l} ACF=\frac{\cos(\varphi_{L2}-\varphi )}{\sin\varphi }=\frac{\cos\left[c(\tau_{L2}-\tau )\right]}{\sin\left(2\pi f_{i}\tau \right)}\\ \tau_{L2}=nL_{2}/c \end{array}$$

**Figure 8:**
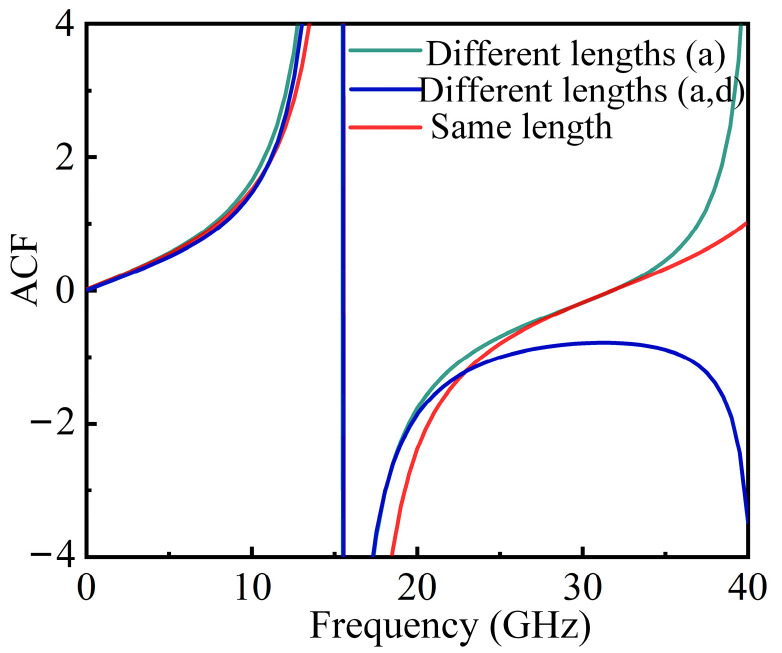
Comparison of ACF curves with different path lengths.

At this point, although the tangent function cannot be directly obtained, the ACF curve is still a monotonic curve, so IFM can be implemented. To prove this point, a set of simulations is conducted. We set the delay to 15 ps. When the lengths of all four paths are identical, the ACF curve is shown as the red line in Figure 8. When paths b, c, and d (paths a, b, c, d are shown in Figure 1) are the same length, and path a is 1 km longer, the ACF result is represented by the green line in Figure 8. When paths b and c are the same length, and path a is 1 km longer, while path d is 2 km longer, the ACF curve is shown as the blue line. It can be observed that a discrepancy in the length of just one path does not affect the ACF results. However, when the lengths of two paths are inconsistent, the measurement results at high frequencies are impacted. Therefore, in the experiment, the path lengths should be kept as consistent as possible. Additionally, factors such as temperature changes and environmental vibrations can introduce additional phase shifts in the fiber, thereby affecting measurement results. Since the length of the fiber contained within the MZI structure in the system is relatively short, the impact is minimal. Moreover, the use of phase-stable optical fibers and integrated systems in the experiment helps to reduce environmental influences.

### 4.2. Scheme Improvement

**Figure 9:**
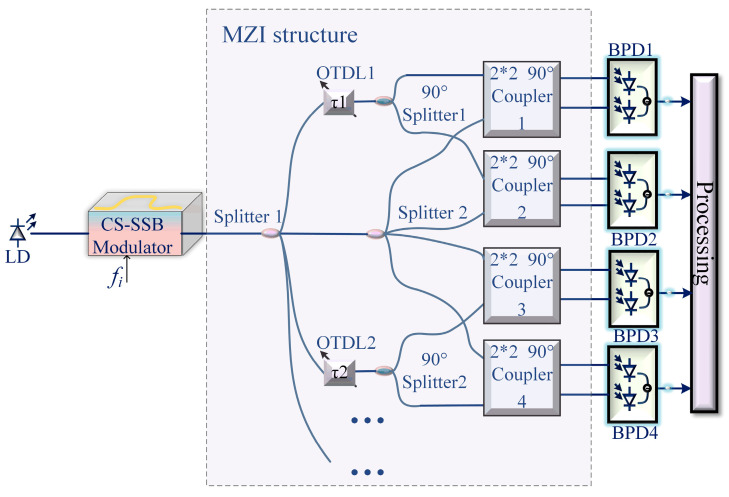
Multi-channel schematic diagram.

The smaller the delay time in the proposed scheme, the wider the bandwidth of IFM without ambiguity. In order to improve frequency measurement accuracy, it is necessary to increase the delay time. However, the corresponding reduction in the unambiguous bandwidth results in a contradiction between frequency measurement accuracy and operating bandwidth. Therefore, in order to solve this contradiction, multi-channel parallel deblurring technology can be used. The longest delay line determines the frequency resolution and frequency measurement accuracy; the shortest delay line determines the operating bandwidth. The improved multi-channel schematic is shown in Figure 9. In the improved scheme, two-channel or multi-channel delay is used, different ACF curves are obtained by adjusting different delays, the approximate frequency of the SUT is obtained by the small delay, and more accurate measurement results are obtained by the large delay path, thus improving the measurement accuracy.

**Figure 10:**
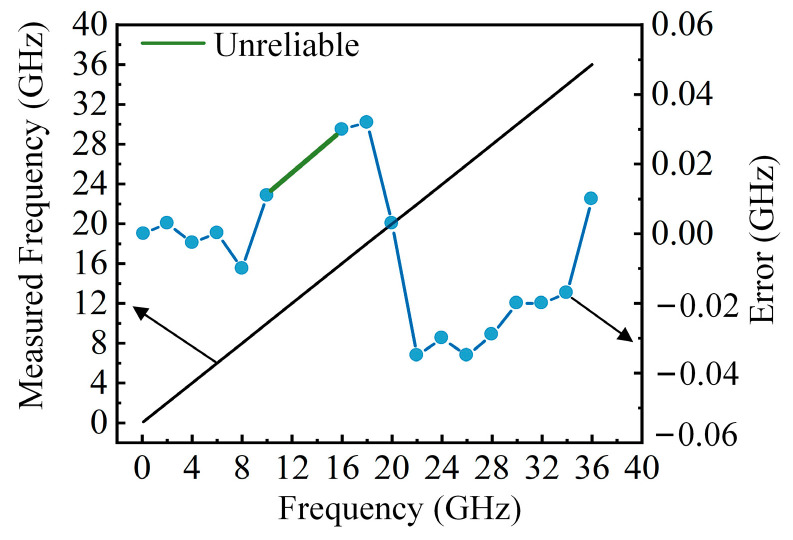
Frequency measurement results and measurement errors with a delay of 20 ps.

Taking two channels as an example, OTDL1 in channel 1 is set to 10 ps, and the measurement results are shown in Figure 4. In channel 2, OTDL1 is set to 20 ps; the measurement results and errors are also tested. The SUT signal is changed from 0.1 GHz to 40 GHz with a step of 2 GHz. The ratio of the output amplitudes at each frequency is calculated and compared with the ACF curve to obtain the measurement value and error; the results are shown in Figure 10. The frequency measurement range is approximately 0.1–10 GHz and 16–36 GHz, and the measurement error is between −0.035 and 0.035 GHz, as shown by the blue dots in Figure 10. A maximum relative measurement error of 0.18% is obtained across the entire frequency range. By combining Figure 4 and Figure 9, more accurate measurement results can be obtained in a large frequency range of 0.1–40 GHz, and at the same time, the delay can be further increased, thus improving the measurement accuracy.

**Figure 11:**
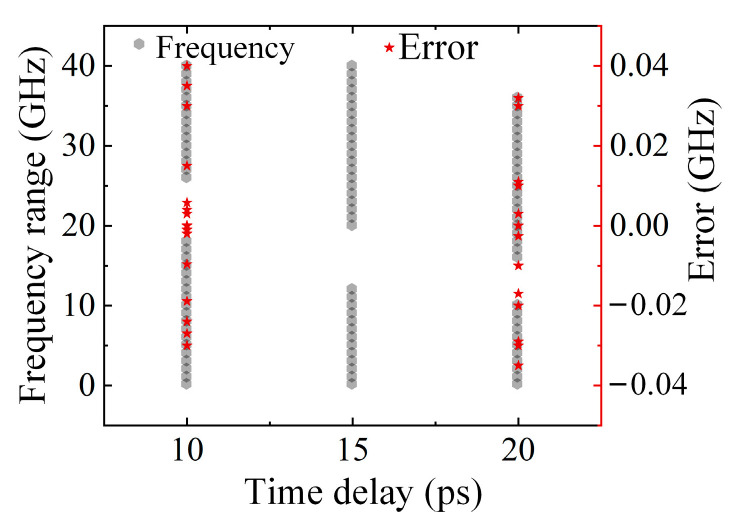
The relationship between measurement bandwidth and error with delay variation.

The relationship between the unambiguous frequency bandwidth, unreliable interval, and error range as a function of delay is shown in Figure 11. As can be seen from the figure, different delay times have different measurement ranges and errors, and the delay time can be flexibly adjusted according to demand in practice.

### 4.3. Long-Distance Measurement

**Figure 12:**
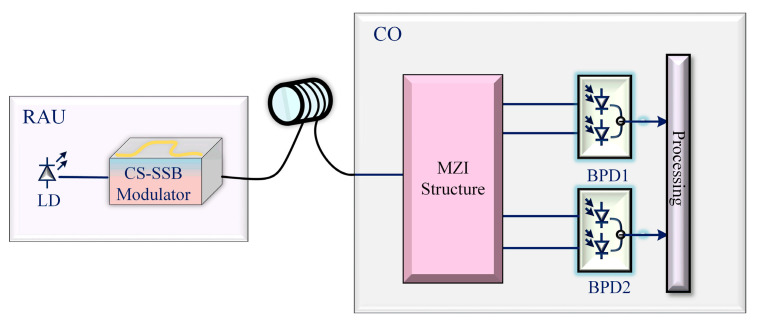
Schematic diagram of long-distance measurement.

As is well known, periodic power fading caused by dispersion in single-mode fibers (SMFs) can be avoided through single-sideband transmission [32]. In electronic reconnaissance applications, microwave receivers face the danger of being detected, and the distance between the radio access unit (RAU) and the central office (CO) of most measurement systems is close, at which time, the entire measurement system may be destroyed. Due to single-sideband modulation, periodic power fading is suppressed in the proposed system, and RAU can be effectively isolated from CO through long-distance fiber transmission, as shown in Figure 12, which has great prospects in electronic reconnaissance applications.

**Figure 13:**
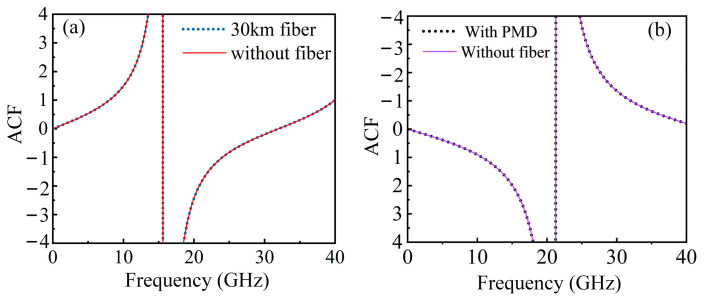
Comparison of ACF Curves (**a**) without PMD (15 ps) (**b**) with PMD (10 ps).

To verify the long-distance transmission performance of this system, a simulation is conducted according to the structure shown in Figure 12. The length of the single-mode fiber (SMF) is set to 30 km, and the OTDL is set to 15 ps. The resulting ACF curve, compared with the curve without the fiber, is shown in Figure 13a. As can be seen from the figure, the ACF curve obtained after long-distance transmission is completely consistent with the one without the fiber. In order to verify the measurement performance of the high-frequency signal at this time, ten sets of measurements are conducted on a 40 GHz signal, with measurement errors less than 0.03 GHz, indicating that the system is capable of the long-distance transmission of high-frequency signals. To further verify the impact of polarization mode dispersion (PMD) on the system, a larger PMD value (5/31.62 (ps/m^1/2)) is added based on 30 km fiber transmission while changing the delay to 10 ps to test the tunability of the system. The results are shown in Figure 13b. In Figure 13b, the purple line represents the ACF result without fiber, while the black dashed line indicates the ACF curve under the condition of the 30 km fiber transmission that includes PMD. It can be seen that PMD does not affect ACF construction during long-distance transmission.

### 4.4. Carrier-Suppressed Single-Sideband Modulation Experiment

**Figure 14:**
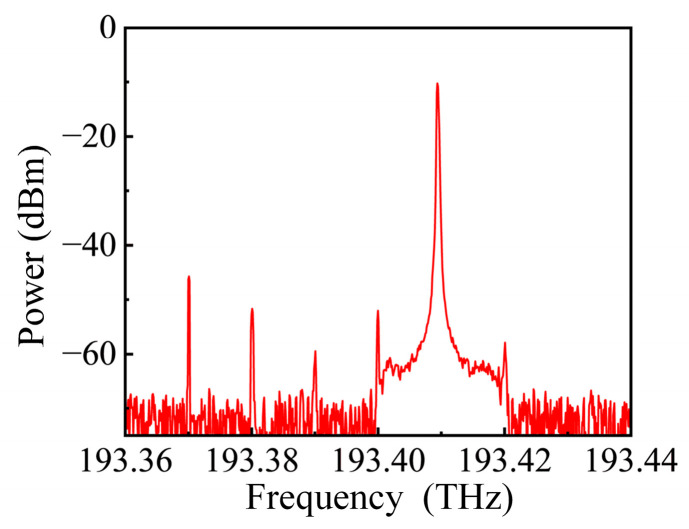
The output spectrum of the modulator.

The CS-SSB modulation of the signal is a critical part of this system. Therefore, experimental validation is conducted on the CS-SSB modulation. In the experiment, a laser (Febo Optoelectronic (Shanghai, China), TLS-M-C-16-P-FA) is used to generate an optical carrier with a central frequency of 193.4 THz and a power of 14 dBm. The optical carrier is transmitted to a DPMZM (Fujitsu (Tokyo, Japan), FTM7961EX), which has a bandwidth exceeding 30 GHz. An RF signal source (Ceyear (Chengdu, China), 1435F) is used to generate a 10 GHz signal, which is then connected to a 90-degree coupler and divided into two paths, transmitting to the two sub-modulators of the DPMZM, as shown in Figure 1. An optical spectrum analyzer (Finisar (San Jose, CA, USA), WaveAnalyzer1500S) is used to analyze the spectrum. The spectrum measured after the modulator is shown in Figure 14, where it can be observed that the carrier–side ratio reaches nearly −40 dB, which is consistent with the simulation results in Figure 2a. This alignment of simulation parameters with actual experiments allows for a more accurate simulation.

### 4.5. Scheme Comparison

Finally, this scheme is compared with other typical schemes, and the results are shown in Table 2. It can be seen that this system can achieve a large measurement range, and the power is tunable, which is not mentioned in most schemes. This also demonstrates the advantages of the proposed system.

## 5. Conclusions

In order to overcome the limitations of traditional microwave IFM measurement, such as limited frequency range and electromagnetic interference, a microwave photonic IFM scheme is proposed in this paper. In this scheme, an ACF curve is constructed by the ratio of two signal amplitudes, and multi-channel delay can be introduced, which can realize high-precision measurement with a large and unambiguous bandwidth. The simulation results show that the proposed scheme can achieve high precision measurement in a wide frequency range of 0.1–40 GHz, the measurement error is −0.03 to 0.04 GHz, and the tunability of the SUT power is verified. The proposed scheme has great application prospects in electronic reconnaissance and communication.

## Figures and Tables

**Table 1:** Simulation parameter settings.

Device	LD	DPMZM	ODTL	90° Coupler	BPD
Reference devices	Feibo Optoelectronic (Shanghai, China) TLS-M-C-16-P-FA	Fujitsu (Tokyo, Japan) FTM7961EZ	Ziguan(Sichuan, China), ODTL	Kylia (Paris, France) COH24	Discovery Semiconductors (New Jersey, USA) DSC740
Device website	www.fiber-photonics.com	www.fujitsu.com	www.zg-photonics.com	www.bonphot.com	www.discsemi.com
Main parameters	Center wavelength: 1527–1668 nm Power: 16 dBm RIN: −155 dBc/Hz	Half-wave voltage: 3.85 V@10 GHz 4.3 V@20 GHz 4.67 V@30 GHz 4.94 V@40 GHz; Insertion loss: 8 dB	Delay range: 0–700 ps Insertion loss: 0.8 dB	Insertion loss: 1 dB Amplitude/phase error: 0.5 dB/<5°	Responsivity: 0.6 A/W; Amplitude/phase error: 0.5 dB/3°

**Table 2:** Comparison of IFM schemes.

Method	Bandwidth (GHz)	IFM Error Within	Tunable Power
[19]	2–19	±0.2 GHz	Not mentioned
[20]	0.5–40	±0.5 GHz	Not mentioned
[21]	1–20	±0.1 GHz	Not mentioned
[22]	0.04–40	0.1 GHz (0.35%)	51 dB
[23]	1–10	±0.2 GHz	Not mentioned
[24]	8–18	±0.15 GHz	Not mentioned
[25]	7.5–20	0.1 GHz	Not mentioned
[26]	5–20	5%	Not mentioned
[27]	20–36	±0.4 GHz	Not mentioned
[28]	1–40	0.2%	Not mentioned
[29]	4.4–8.7	±0.2 GHz	Not mentioned
[30]	0–14	0.075 GHz	Not mentioned
This work	Over 30	−0.03–0.04 GHz (0.3%)	Over 30 dB

## Data Availability

Data are contained within the article.
